# Supplementary material for: The influence of sexual arousal on subjective pain intensity during a cold pressor test in women
Source: PLoS One. 2022 Oct 5;17(10):e0274331. doi: 10.1371/journal.pone.0274331 (PMC9534408; doi:10.1371/journal.pone.0274331)
Supplement: Attachment — Submitted filename: Response to Reviewers.docx [file pone.0274331.s002.docx]

Dear Dr. José A Hinojosa,

We would like to thank you, as well as the reviewer, for the time you have dedicated to our manuscript. We appreciate the constructive and helpful comments, and we gladly accept your invitation to revise and resubmit our manuscript (PONE-D-22-05538) titled "The influence of sexual arousal on subjective pain intensity during a cold pressor test in women". Below, we have detailed our responses to your comments and suggestions. We hope that the revised manuscript now qualifies for publication in PLOS ONE, and we look forward to hearing back from you.

With warm regards,

Lara Lakhsassi, on behalf of the co-authors.

# Review Comments to the Author:

| General comments:   1. It would be convenient for the authors to share their database during the review process, beyond that, once published, they can share their data. 2. It would be useful to edit the figures to make them easier to understand.- 3. To facilitate the review process, it would be convenient to include line numbers on the manuscript pages. | Author response:   1. We have created a space on DataverseNL in which we have uploaded our data, and we will make the data available to you as soon as anonymity has been reviewed and ready to publish. [[https://doi.org/10.34894/BRZT3O](https://doi.org/10.34894/BRZT3O), DataverseNL, DRAFT VERSION] 2. In line with your suggestion, we have edited the figures to make them easier to understand. Instead of a boxplot, we have created a simpler presentation of the means with error bars (95% CI). 3. In line with your suggestion, we have added line numbers to the manuscript pages. |
| --- | --- |
| Introduction:   1. In general, the introduction gives a coherent review of the state of the art, gathering sufficient literature on the subject. 2. An "Introduction" heading at the beginning of the section is necessary. 3. Specific hypotheses on the expected effects should be included in the final part. | Author response:   1. Thank you, we are happy to read that the introduction provides a solid review of the literature. 2. We have added a heading, as suggested   .   3. In line with your comment, we have amended our hypotheses in the final part and are now more explicit with regard to the expected effects. The relevant section now reads as: *In short, we hypothesized that: (i) participants in the sexual arousal condition would report less pain during the CPT than participants in the neutral condition, (ii) participants in the sexual arousal condition would also report less pain than those in the mere distraction and general arousal conditions, and (iii) that, in line with previous research, participants in the distraction and general arousal conditions would report less pain than those in the neutral control condition [16-19].* |
| Method:   1. The method presents the information scattered throughout the section. My recommendation is to make fewer sub-sections and to integrate information about the experiment, as well as the measurements and stimuli used, in the "Procedure" section. 2. Were differences in pain thresholds between participants assessed in any way? This would be important to do as the pain experience is highly subjective and controlling and then manipulating it is one of the key elements when working with pain. If different groups per se have different pain thresholds, this could influence subsequent outcomes. If this was not controlled for, it would be necessary to justify why. 3. Was the choice of the videos used based on any kind of pre-assessment or was it randomly selected? 4. Sexual orientation and age characteristics are assessed with questionnaires, but these data are not reflected in any section afterwards. A table with this information could be included at the beginning of the results. 5. Only one measure of pain was taken during the video?. It would be more convenient to take several pain measurements to generate the threshold. As I mentioned before, there are many variables that affect the perception of pain, and several measurements could reduce this influence. 6. The section "Data Analyses Plan" should be renamed as "Data analyses" or something similar. In this section there are again too many sub-sections with fragmented information. My recommendation, again, is to integrate the information to make it easier to follow. It would also be useful to integrate the information on the software used into the text. 7. The subsection "Hypothesis Testing" could be deleted or at least integrated into the previous ones. 8. The abbreviation CPT is used for the first time in this section but has not been previously defined. | Author response:   1. In line with your recommendation, we have reduced the amount of sub-sections in our manuscript and have integrated some of these sections (i.e., "E-prime software", "questionnaires", together with procedural aspects of the measurements) into the "procedure" section. We have additionally shortened the headings in the "Measures" section (e.g., see "manipulation check" and "pain") to make them more concise. 2. Consistent with previous research using a cold pressor test to elicit pain, we used a fixed water temperature that was similar for all participants. We did not assess individual differences in pain thresholds before the experiment. However, participants were randomly allocated to one of the four experimental conditions. Thus, although individual differences in pain threshold may have influenced subjective pain and the time participants kept their hands in the CPT, such influence would be independent of condition.   Nonetheless, it understandably cannot be ruled out that perhaps, by chance, some a priori differences between conditions might have been present, in turn reducing the sensitivity of the design to find differences in subjective pain between conditions. Unfortunately, this cannot be checked, and we have therefore now mentioned it in the discussion section as a limitation. The added text reads as follows: *Finally, although a fixed water temperature was used across experimental conditions, and random allocation to experimental conditions was utilized in order to prevent an unequal spread of individual differences in pain thresholds between conditions, it cannot be ruled out that perhaps some a priori differences between conditions might have been present, in turn reducing the sensitivity of the design to find differences in subjective pain between conditions. Future studies may consider implementing a baseline pain threshold measurement in order to check whether this may have occurred.*   3. The choice of the neutral film was selected based on a series of previous studies (e.g., Borg & de Jong, 2012) in which the train film was successfully used. The general arousal and sexual arousal films were carefully selected by the research team upon consensus that these will potently elicit sexual or non-sexual arousal. The manipulation checks have confirmed the validity of the selected films.   We have now mentioned the selection process in the "film stimuli" section, reading as follows: *The choice of films used for eliciting (sexual) arousal (i.e., porn and parkour films) were selected by the research team upon consensus that these would successfully elicit the target emotional states. The choice of the film used to elicit a neutral or distraction state was selected based on a series of previous studies within our research group in which the train film was successfully used.*  In addition, we have changed the words "carefully piloted" to "selected" in line 1041.   4. We have now added the means and standard deviations relevant to age and sexual orientation in the Methods section, under 'Participants'. Given that this section is where we first describe our sample, we thought it would be more appropriate to include these descriptives here, rather than in the Results section. The two paragraphs in the Participant section have additionally been integrated into a single paragraph. 5. To clarify, indeed only one measurement of pain was taken, but this was done after the video at the time the person had removed the hand from the CPT. Although we agree with the reviewer that there are clear benefits for using multiple pain assessments, our decision to nevertheless rely on a single measurement of subjective pain was based on having as few interruptions during the film as possible such that the participant may fully emerge in the critical motivational state which could be compromised by repeated assessments. Although this approach may come at the cost of rendering the measure more sensitive for (uncontrolled) variables that may affect pain perception, this seems not critical for the internal validity of the design as any factor that would affect pain apart from the particular condition (i.e., experimental manipulation) would have an affect independent of condition. 6. In line with your suggestion, we have renamed the "data analyses" section. We have additionally integrated the information and reduced the amount of sub-sections (e.g., integrated the two types of manipulation checks (MC) – "MC prior to CPT" and "MC of experimental procedure" – under one heading titled "manipulation checks"), as well as added the software used in the text. Accordingly, we have integrated the corresponding sections in the 'Results' section as well to maintain consistency. 7. We have integrated the 'hypothesis testing' section along with the 'assumptions' section. 8. Thank you for making us aware of this error, we have now defined it in the introduction section where we introduced the task (and used the abbreviation throughout the remainder of the text. |
| Results:   1. The use of the same terms to refer to dependent variables and conditions is confused. This makes the reading of the results confusing and makes it difficult to follow the results. I advise using different names for both. 2. Effect sizes are only reported for some analyses, it would be interesting to report them for all analyses. 3. In general, the description of the results is complex to follow, perhaps because of what I said before about the naming of variables. I would revise the wording to make it clearer. 4. In the last section of the results, an analysis is made excluding certain subjects. In my opinion the exclusion of these subjects is arbitrary, although the authors explain that it is a technique comparable to that based on mean scores. I believe that an exclusion strategy based on outlier analysis would be more convenient and could shed more light on the data. | Author response:  1. We understand that using the same terms can elicit confusion. In response to your comment we have therefore edited the phrasing to detail the three conditions as: SEX condition, PARKOUR condition, COUNTING condition, and TRAIN condition. These labels have now been introduced in the Methods section, under 'Manipulation Check', and used to refer to conditions thereafter. The dependent variables in the Results section will read "sexual arousal", "generalized arousal", and "distraction".  2. In line with your suggestion, we have now reported all relevant effect sizes across all analyses in the Results section.  3. In line with your recommendation we have revised the wording for the variables and conditions to make it easier to follow.  4. In retrospect, we agree that the exclusion of these subjects can be considered as arbitrary. Also, a data-driven strategy might be considered arbitrary/problematic when there are no reasons to suspect that the data-points are invalid. We therefore eventually decided to restrict from further post hoc analyses and to remove the "sensitivity analyses after post hoc exclusions" that was part of the original submission. |
| Discussion:   1. The discussion is sparse and does not allow for a detailed explanation of the main results. Much space is devoted to commenting on the results, but there is no explanation or comparison with other articles of the most relevant results, i.e., the null effect of sexual arousal on pain perception. In the limitations, some data are given on why sexual arousal may be perceived differently in women, but this information should be discussed in more depth in the corresponding part of the discussion. 2. The first two paragraphs of the discussion could be integrated into a single paragraph in which only a summary of the results found is presented, followed by a paragraph discussing the most relevant results. 3. In general, there is little bibliography in this section (there are only 6 references in the whole discussion). A more extensive literature search is needed to discuss the results found. | Author response:  1. We have restructured the discussion in line with your suggestion. We have accordingly added comments on the results and a comparison to previous findings. The added discussion read as follows: *While the findings in this study corroborate previous research showing that subjective sexual arousal per se is not sufficient to elicit robust analgesic effects in women, it has also been hypothesized that the lack of a pain modulatory effect may be attributable to female participants having experienced feelings of disgust alongside sexual arousal [13]. Indeed, women are substantially more likely to respond to pornography with negative affect such as disgust [13, 14, 24-26]. Germane to this, it has been found in previous research that for women, pornographic stimuli elicited similar brain responses as prototypical disgusting stimuli. Moreover, it has been found that the brain responses to penile-vaginal penetration pictures were modulated by the level of subjective disgust elicited by these sex stimuli. [27, 28]. If indeed such avoidance-related affective states were elicited by the current SEX condition, these might have counteracted the influence of approach-related appetitive affective states (e.g., sexual arousal) resulting in exacerbated pain [29], rather than inhibited pain. In other words, if indeed disgust was elicited along with sexual arousal, this might have counteracted the pain reducing effects of sexual arousal and thus nullified the net effect of the manipulation on pain. Future studies might therefore consider attempting to minimize this risk by critically assessing the pornographic stimuli used for female participants such that they elicit as little aversive affect as possible. Accordingly, studies show that female-centric pornography – typically involving a lengthier duration of foreplay and an emphasis on female pleasure – significantly increases subjective sexual arousal in women in comparison to male-centric "commercial" pornography whilst minimizing aversive affect [14, 25, 26]. Taken together, while the current study adds to the available evidence that subjective sexual arousal per se is not sufficient to elicit robust analgesic effects in women, we cannot exclude the possibility that no effect was found in women due to (concurrent) negative affect associated with the pornographic stimulus. In future studies, an assessment of negative affect during the experiment, alongside an implementation of female-friendly stimuli, is recommended as a potentially helpful means of (statistically) controlling for disgust when attempting to induce sexual arousal in women. In the event that the implementation of these recommendations continue to show no pain modulation in women during sexual arousal, follow-up studies would be necessary to test whether this conclusion still holds with alternative sex stimuli. For instance, earlier studies show that direct tactile stimulation can successfully elicit sexual arousal to initially neutral stimuli [30]. Such an approach may also have the advantage that it minimizes the chances of inadvertently eliciting negative affect like disgust, and may therefore be especially effective in assessing the impact of sexual arousal on pain in female participants.*  2. In line with your suggestion, we have integrated the two paragraphs into a single paragraph summarizing the results of the study. This is followed by a paragraph discussing/contextualizing these results (as detailed in point 1).  3. In adding further discussion of the results, we have expanded our bibliography further. In addition, we verified/assured (via extensive literature searches) that all studies that are most critical/related to the influence of sexual arousal on pain in women were covered in our introduction and discussion sections. |
